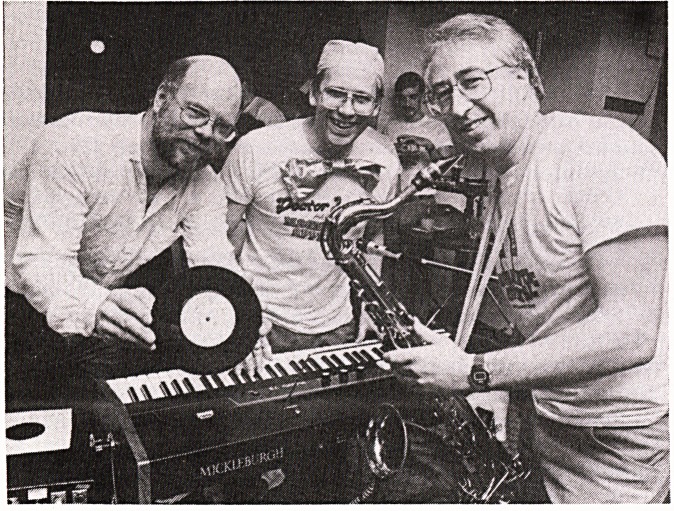# From Our Correspondents

**Published:** 1986-06

**Authors:** 


### From Our Correspondents

### Princess Anne visits the Bristol Radiotherapy Centre

On the 6th March the Bristol Radiotherapy Centre had the honour of a visit by Her Royal Highness Princess Anne. The Princess was there to officially open the new Linear Accelerator made by Varian Associates of California. The machine will be particularly useful for the treatment of breast cancer, certain gynaecological cancers and the tumours of pelvic origin. Very precise location of the tumour volume is required for these radical treatments and the new accelerator is well suited for this work.

*Fig.* 1. Princess Anne receiving a bouquet of flowers from Richard Cottey. Pictured with them is Richard's doctor, Dr Jill Bullimore (Radiotherapist). In the background are Mrs. Barbara Sholl-Evans and Mr. Charles Clarke.

### Record Released to help the Bristol Magnetic Scanner Appeal

A group of local doctors and NHS staff have produced a record to help the Magnetic Scanner Appeal. The group is known as the Doctor Jazz Quartet; pictured holding the record is the group's drummer, Chris Chivers, who is a paramedic. Hugh Coakham (Frenchay Neurosurgeon) is shown with his saxophone and Paul Goddard (BRI radiologist) is at the piano.

The A-side of the record is called 'Magnetic Appeal' and the B-side Transplant Surgery' – subtitled 'a man after my own heart'. The record is on sale for £1-50p from the X-ray department and HMV and other record shops and has already gone to a second pressing.

The piano shown here was provided on 'permanent loan' by Mickleburghs, for the band's use at fund-raising events. Another recent and very generous donation came from the friends of Ham Green Hospital. They have raised £1,000 for the appeal.

The White Lion public house, Frenchay, has raised over £6,000 and the Hambrook Inn has raised close on a thousand pounds.

Mr. John James has kindly donated one million pounds to buy the Magnetic Scanner for the N.H.S. unit and it will be the first N.H.S. unit in the South West.

The Bristol M.R.I. Scanner Fund would welcome any assistance that can be given to help them to raise the necessary funds to erect the building and run the Scanner. Please contact either Dr. Paul Goddard (Bristol Royal Infirmary) or Mr. Hugh Coakham (Frenchay Hospital).

P. R. Goddard

### Charles Clark Retires

An illustrious chapter in the history of the Bristol & Weston Health Authority and formerly the United Bristol Hospital has closed with the retirement of Mr Charles Clarke.

Mr Clarke has been a tower of strength for the Health Service for many years. After war-time service in the Welsh Guards and a career in his family firm of solicitors, Mr Clarke soon showed that he had inherited his father's concern for the Health Service. Initially serving on committies for the Homeopathic Hospital, he followed this as Chairman of the Homeopathic Hospital from 1960 and chairman of the Board of Governors of the Bristol Royal Hospitals in 1968 and latterly of the Bristol & Weston Health Authority.

Many changes have arisen during his time as Chairman and advances have occurred every year. Recent successes have included the opening of the Bristol Eye Hospital and refurbishment of the Dental Hospital. Major projects underway include the building of the new Weston Hospital and the recent announcement of new plans for the Bristol Royal Infirmary.

Charles Clarke has guided all the changes with a firm hand on the tiller and with sound judgement. He has always been approachable and his kindliness to the hospital staff and patients is well known.

P. R. Goddard

### The New Chairman of Bristol and Weston Health Authority

Following the retirement of Mr Charles Clarke a new chairman has been appointed to Bristol and Weston Health Authority. He is Mr. Peter Durie, aged 60.

Mr Durie has had two previous, highly successful careers. From the age of 18 to the age of 38 he served in the army in the Artillery and as a parachutist. He retired from the army as Lieutenant Colonel and his many distinctions included the M.B.E. and the George Medal.

His second career was in the brewing world with Courages. He had various appointments including Assistant Managing Director of the group. He moved to Bristol in 1969 and has lived in Wrington ever since.

Mr Durie has been a member of the management board of the Royal Hospital and Home for Incurables, Putney.

Mr Durie is a very active man and engages in much outdoor sport including skiing, tennis, sailing and wind surfing. When in a more contemplative mood he particularly likes classical music and gardening. He is married with adult children and grandchildren.

Peter Durie has been appointed as Chairman of the Bristol and Weston Authority for a 4 year term and we wish him all success.

P. R. Goddard

**Referrals**

Avon Local Medical Committee recently discussed the ridiculously long orthopaedic waiting list – in one district about one year for routine referrals. Many possible reasons were put forward including 'unnecessary' referrals being made by general practitioners, consultants spending too much time with private patients, limited operating time, increased orthopaedic work load and so on. It was difficult explaining the long waiting period when some other districts had substantially reduced theirs by relatively simple procedures like seeing more patients per session or employing a clinical assistant.

General practitioners refer on average eight patients per 100 consultations to other physicians. This figure hides considerable variation – one recent study showed general practitioner referral rates from 2 to 25 per 100 consultations. In 1983 it was estimated that each outpatient consultation costs about £15 but, of course, this figure is only part of the cost of referral. Klein talks about the general practitioner being the 'gatekeeper to the NHS' and it is this factor that causes concern, not the simple matter of referral; the real costs of referral come after the outpatient consultation. Whether a patient with a backpain or a bunion or a recurrent sore throat gets referred is determined by an immensely complicated series of factors. What happens after referral is also very variable but the cost implications of these later decisions are of a much greater magnitude.

Let us examine the factors that are important in deciding whether referral is made. They can conveniently be divided into two groups: patient-related variables and physician-related variables. The former relates to matters such as the seriousness of the patient's complaint, the age and social class of the patient, the distance of the patient from the hospital, the ability of the patient to pay for private consultations and the pressure from the patient or his relatives or friends for referral. The physician-related factors are also complex and include factors such as the doctor's training and experience, the availability of facilities for investigation and treatment, the doctor's ability to tolerate uncertainty and to evoke confidence in his patient and his previous experience of the effects of similar referrals.

It is well recognised that different general practitioners have different 'referral thresholds' and most partnerships are aware of different thresholds within their practice. One doctor may refer more patients to a particular specialty either because he is more uncertain of management possibilities or he is aware of the possibility of a greater range of therapeutic interventions than his colleague.

One other important factor is the local 'conventional wisdom' regarding particular referrals. Statements such as 'it is useless to refer such a problem' soon becomes accepted as a rule as does a particular physician's superior ability to manage a particular disease.

The complex interactions that go on between patient and doctor in the referral process are only just being teased out; much more needs to be done before we can begin to say that we understand completely this fascinating interaction.

M. J. Whitfield

**Paying for it all**

*Question* What is the current revenue budget of the SWRHA?

*Answer* £600 million for a total population of 3.1 million

How do I know? Because I found it in the booklet '10 year plan for better health' attractively produced by the RHA to summarise its strategy for the next decade. Before some 'Clever Dick' says 'can't they find something better to spend their money on' can I suggest that, in its absence, the same individual would complain that 'they don't tell us anything.' I recommend that you get a copy.

Of course, every one of us will find good reason why the money should be spent differently, but I think the penny is beginning to drop that money doesn't grow on trees. By spending more on A we inevitably have less for B (and probably C & D also). This is the first message we must get across to our 'consumer' (what a ghastly word!). The second is even more important; namely that a significant proportion of the £600 million is spent on dealing with conditions which result directly from the life-style or working and home environment of individuals and families. Among these I would include smoking, (active & passive), bad nutrition, alcohol and drug misuse, dental caries, environmental hazards in home and workplace, road traffic accidents (especially motorbikes), cardiac disease, cervical cancer, S.T.D.s and many unwanted pregnancies. The necessity to screen all blood for A.I.D.S. can also be included.

Thus when so much money is being spent on retrieving the consequences of personal selfishness, greed and stupidity, no wonder we cannot afford to care adequately for the very young, and the very old, and the mentally and physically handicapped.

The ten commandments may be unfashionable, but they are still good public health. That is why it is good to see that 'promotion of good health and disease prevention' is the first key commitment in the strategic plan. Let's get on with it.

G. M. Stirratt

**Care of the elderly**

A recent visit to West Germany emphasized to me how much further forward we are in this country in terms of developing services for the elderly than is the case in other parts of the world. The development of an integrated approach to Geriatric Medicine is extremely patchy. In Germany, as most readers will know, however, their health services are organised very differently to ours, and in my opinion our own National Health Service is far superior. It is a tragedy that consciously or subconsciously the present 'administration' is allowing so many aspects of it to deteriorate.

Colleagues will probably like to know that Dr. John Pounsford, currently Lecturer, has been appointed to the Senior Lecturer's post in our Department. This brings us back up to our full complement of consultants. Dr. Roy Holman, who has been with us for six months as a Temporary Lecturer, has obtained a definitive Senior Registrar post in Bath, to which he will be going at the beginning of May. Congratulations to both of them, and also to Dr. Peter Murphy, who has recently been appointed as the third consultant in the Bristol & Weston District, to work with Bill Lloyd and Angus Windsor. He is a Senior Registrar in Cambridge, and will be joining us in the West Country in the near future.

Dementia may seem tedious to most, and an insoluble problem to many of us. Despite this, the Memory Disorders Clinic at Manor Park Hospital tries to provide a thorough multidisciplinary screening and management approach for those referred. I only mention this because we have been surprised to discover that about a quarter of the patients we have seen are probably not suffering with dementia! As they were all referred with this diagnosis having been made, either by their family doctor or a hospital clinic, it just goes to show how difficult it is to evaluate intellectual impairment.

Finally, non-apiarists might be interested to know that in my part of the world this has been a bad winter for our bees. Paradoxically, not because of the cold spell, but because the longer period of milder weather, albeit very wet, during which the bees were more active than usual within their hives. This has resulted in a greater consumption of winter stores than would have been the case if we'd had uniformally low temperatures, encouraging a tighter cluster with less activity within the hive.

G. K. Wilcock

**They talk differently in other languages**

People have different ideas about the best way to to talk to one another. When baffled by such conversational differences in this country we sometimes say that someone is talking Double Dutch. What we mean is not that Dutchmen are especially odd, but that our particular companion of the moment, usually an Englishman, is proving less than easy to follow.

Pastimes can lead to unexpected situations. There is nothing more safe, English and predictable than wanting to play cricket. Visions of summery village greens, conservative middle-aged men dressed in a mixture of sports jackets, cavalry twills, grey or cream flannels and the occasional multicoloured blazer or neckerchief easily spring to mind. All very dull, predictable and English.

All English that is unless you happen foolishly to be doing the same thing elsewhere, like in Holland. The scene is very familiar: rain on the way, sports jackets, and even more blazers and club ties than are usually seen anywhere here in this country. It all looks rather like the sepia-coloured photographs of the 1930's hanging in the Hampstead Cricket Club pavilion. Not only that, but the beer has a mouth-curdling feel which you cannot remember since first drinking illicitly under age in a corner of the same Hampstead club house.

Anyway, there you are quite at home in Holland. The chat is all the same; the umpires are no different, and the matting wicket plays the same unfair tricks that any grass pitch does here. In the end it matters little who won the game, more or less. So far so good.

Dutch people have an extraordinary friendliness for the English. This shows in almost every possible manner. Losing your way, your passport or even your money will bring out the very best in all Dutchmen. No English visitor could possibly complain, or feel other than entirely welcome. Even the police are not as threatening as elsewhere on the continent.

Then comes the downfall. Total surprise. You are after all in a different country. It seems only polite at breakfast time to try out a few newly learnt words to show your hosts that you are not only grateful for their kind hospitality, but are trying to say the odd word in single, rather than double Dutch.

I don't recommend it. Do by all means try Dutch on your first visit, but alone in the bathroom, preferably with the bath water running and the radio on at full volume.

There are some Dutch words which a prudent Englishman can say with impunity in Holland. Alas, these are not only few but they are mostly monosyllables. On their own they tend not to lead to sparkling conversation. The most dangerous conversational topic is an account of where you have been the day before. All French scholars will know of the dangers in risking the pronunciation of '*grenouille*' when otherwise unprepared. French frogs resemble a similar but unsuspected pitfall in Holland. It is fatal to have been anywhere within fifty miles of a place called Scheveningen. You and I might think that this was just a pleasant sea-side resort with mild maritime breezes, frequented by anyone with a certain boredom for Bournemouth, Cannes or Torquay. It is not. It is a death trap.

Dutch families are like most English families. Early morning is early morning. Gentle doziness and slow awakening take place without any undue fuss. Clocks tick away quietly to everyone's satisfaction. Breakfast is a peaceful time in a sensible family. No one, of sense, says anything controversial. Newspapers, toasting machines and boiled eggs are the order of that time of the day.

However, just let an English guest tell any sleepy family circle at breakfast in Holland that he has been to Scheveningen and it is as if you had suddenly sprayed the place with a magic and mischievous elixir of life. Middle-aged men, even if otherwise respectable neurologists, their previously placid wives, sons, daughters, domestic servants and the domestic pets are given to apoplexy. Tears roll down cheeks. Boiled eggs seem to have gone down the wrong way. Peculiar muffled laughing noises come from places you couldn't imagine. In short it is all bad for the health at that time of the morning. Afterwards everyone is very apologetic. It was not your fault, they say. It is just that Dutch, and especially Scheveningen, are really only for Dutchmen to say.

Where were we? Oh yes, on the way to explain that languages are not the same. Well, of course one doesn't learn. Undeterred by an inability to pronounce, I thought that a quick dip into written Mandarin Chinese[1] might heal old wounds. Messrs. David McKay of New York publish a convenient paperback to suit the purpose. Price: only what you want on most second-hand book stalls.

Dead easy. Best thereafter to avoid Chinese Take-Aways though, just in case. Especially at breakfast time. One would hate to give an unsuspecting Chinaman in the early morning apoplexy or an attack of bean shoots going down the wrong way. It doesn't seem fair, especially since most Chinamen are not given to sporting blazers, or even less of thinking of putting on batting pads.

Thereafter, life seemed to assume an even keel for many years. Any language was fair game, provided that there was no need or excuse to talk it. There were no misunderstandings or unintentional heart attacks caused. No wonder that classical Greek and Latin were once so popular amonst schoolmasters. You can certainly read them quietly at breakfast without giving any offence to anyone in the family.

The mistake? Yes, admittedly there was one. The new language was found quite without knowing that it was even a language. Someone had told me that Word Processors were a good thing. So they are. The trouble is you may then stupidly start to read about how they work. No, not intentionally. But it may happen.

Thus far different languages had seemed to be spoken (or better written) by people who live in different parts of the world. Nevertheless at the back of Word Processors there are other beings who can talk in new and immodest tongues[2]. To begin with they are all capitalized languages. Whoever would admit to talking FORTRAN, BASIC, MALLARD or D BASE II in front of their grandmother? What would happen at breakfast with these strange new computer languages?

Fear not. They may save us all. Who could possibly be upset, even at ten to eight in the morning by reading:

```
600 REM *** add entry ***
601 IF entries ≥ 45 then PRINT, OK
602 WRITE # file %, name
603 NEXT
604 RETURN
605 CLOSE FILE
```

606 REM * change disc *

I doubt if any Dutchman, or even solemn Chinese mandarins at breakfast would turn a hair. I certainly shall not, but then that is not really talking.

607 EXIT.

J. D. D.

**REFERENCES**

1. WILLIAMSON, H. R. (1947) Chinese: basic Mandarin. McKay Publishing, Inc., New York.
2. ILLINGWORTH, V., GLASER, E. L. and PYLE, I. C. (1983) Dictionary of computing. Oxford University Press, Oxford.

## Figures and Tables

**Fig. 1. f1:**
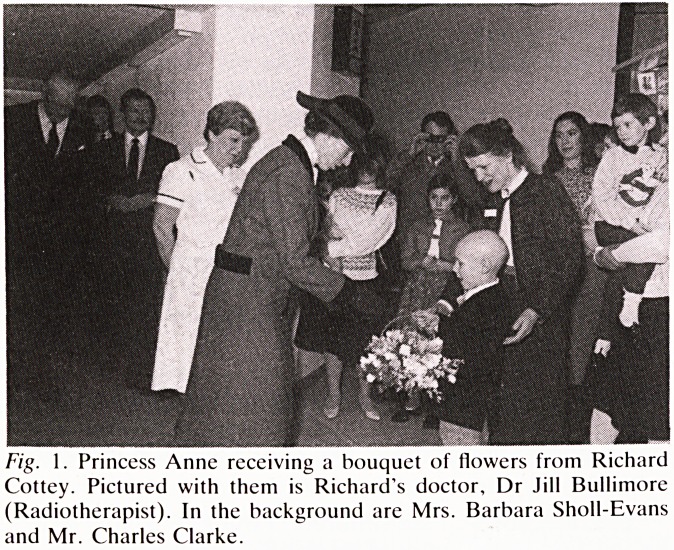


**Figure f2:**